# Bioactive Potential of Actinomycetes from Less Explored Ecosystems against *Mycobacterium tuberculosis* and Other Nonmycobacterial Pathogens

**DOI:** 10.1155/2014/812974

**Published:** 2014-11-09

**Authors:** Radhakrishnan Manikkam, Gopikrishnan Venugopal, Balaji Subramaniam, Balagurunathan Ramasamy, Vanaja Kumar

**Affiliations:** ^1^Centre for Drug Discovery and Development, Sathyabama University, Chennai, Tamil Nadu 600 119, India; ^2^Department of Microbiology, Periyar University, Salem, Tamil Nadu 636 011, India; ^3^Department of Bacteriology, National Institute for Research in Tuberculosis, Chennai, Tamil Nadu 600 031, India

## Abstract

Bioactive potential of actinomycetes isolated from certain less explored Indian ecosystems against *Mycobacterium tuberculosis* and other nonmycobacterial pathogens was investigated. Actinomycetes were isolated from the soil samples collected from desert, coffee plantation, rubber forest, and hill area and their cultural and micromorphological characteristics were studied. Crude extracts were prepared by agar surface fermentation and tested against *M. tuberculosis* isolates by luciferase reporter phage (LRP) assay at 100 *µ*g/mL. Activity against nonmycobacterial pathogens was studied by agar plug method. Totally 54 purified cultures of actinomycetes including 43 *Streptomyces* and 11 *non-Streptomyces* were isolated. While screening for antitubercular activity, extracts of 39 actinomycetes showed activity against one or more *M. tuberculosis* isolates whereas 27 isolates exhibited antagonistic activity against nonmycobacterial pathogens. In particular crude extracts from sixteen actinomycete isolates inhibited all the three *M. tuberculosis* isolates tested. Findings of the present study concluded that less explored ecosystems investigated in this study are the potential resource for bioactive actinomycetes. Further purification and characterization of active molecule from the potential extracts will pave the way for determination of MIC, toxicity, and specificity studies.

## 1. Introduction

Tuberculosis (TB) caused by* Mycobacterium tuberculosis* is a highly prevalent infectious disease with almost one-third of global population believed to be infected [1]. According to statistics, India is the 17th among the 22 high burden countries in terms of TB incidence rates [2]. Emergence of drug resistance among* M. tuberculosis* isolates and long term therapy using combination of drugs for its treatment are the major problem in TB control. Hence, there is an urgent need for new antitubercular drugs to fight against drug resistant* M. tuberculosis* strains [3]. The new anti-TB drugs are expected to have less side effects and improved pharmacokinetic properties with extensive and potent activity against drug resistant strains and/or should be able to reduce the total duration of treatment [4]. Secondary metabolites from microbial sources have a long history in the treatment of TB [5]. Actinomycetes are aerobic filamentous Gram-positive bacteria with true aerial hyphae, belonging to the phylum Actinobacteria (order Actinomycetales) [6]. Actinomycetes are common soil inhabitants with an unprecedented ability to produce clinically useful secondary metabolites including antibiotics. Of the total microbial bioactive metabolites, around 50% are reported from the members of actinomycetes [7]. From the discovery of streptomycin, first antibiotic used for anti-TB therapy from* Streptomyces griseus*, numerous anti-TB antibiotics such as kanamycin and rifampicin have been reported from actinomycetes of terrestrial origin. In recent years, exploration of actinomycetes from routine ecosystems frequently results in reisolation of known actinomycetes and antibiotics. Instead, bioprospecting of un/less explored ecosystems like marine, desert, forests, caves, and hills has been proved as useful method for tapping innumerable number of bioactive compounds from novel bioactive actinomycetes [7–10] including anti-TB metabolites [11]. Bioprospecting of actinomycetes from certain less explored ecosystems in India with special reference to antitubercular activity was attempted in the present work. Antagonistic activity of actinomycetes against nonmycobacterial pathogens was also studied.

## 2. Materials and Methods

### 2.1. Description and Characterization of Actinomycetes

Actinomycetes were isolated from soil samples collected from the rare ecosystems, namely, (i) Thar desert, Rajasthan; (ii) Rubber forest, Kerala; (iii) Coffee plantation, Kerala; (iv) Western Mountain, Thirukurungudi, Tamil Nadu; (v) Thotabeta hills, Tamil Nadu; (vi) Siruvani hill area, Tamil Nadu; (vii) Yercaud hills, Tamil Nadu; and (viii) Munnar hill area, Tamil Nadu. The collected samples were dried at 28°C for 2 d. Actinomycetes were isolated by adopting standard spread plate method using starch casein nitrate agar medium [12] supplemented with nalidixic acid and nystatin to retard the growth of bacteria and fungi, respectively. Growth of actinomycetes was maintained on yeast extract malt extract (YEME) agar (ISP medium 2) [13] as well as in 30% glycerol broth.

Cultural characterization was done by inoculating all the actinomycete cultures into YEME agar medium [13]. Micromorphological characteristics were studied by adopting slide culture method [14]. Based on growth pattern of actinomycetes on YEME agar medium and microscopic appearance, similar actinomycete isolates were discarded and the ones that were exhibiting different characteristics were selected for further investigations.

### 2.2. Small Scale Production of Bioactive Metabolites

Bioactive metabolites from actinomycetes were produced by agar surface fermentation. All the actinomycete cultures were inoculated into two YEME agar plates each and incubated at 28°C for 10 d. After scraping out the mycelial growth, the agar medium was cut into pieces and metabolites secreted extracellularly into the agar medium were extracted using 50 mL of methanol for 24 h. The methanol portion was collected and concentrated using eppendorf concentrator at 30°C and quantified [15]. One mg per mL of working concentration of crude extract was prepared using 10% dimethyl sulfoxide (DMSO) prepared in sterile distilled water and filtered using 0.45 *μ* filters.

### 2.3. In Vitro Screening for Antitubercular Activity

Antitubercular activity of actinomycete extracts was studied against standard laboratory strain* Mycobacterium tuberculosis* H37Rv, SHRE (streptomycin, isoniazid, rifampicin, and ethambutol) sensitive, and SHRE resistant clinical isolates of* M. tuberculosis* by adopting LRP assay [16]. All the* M. tuberculosis* isolates were obtained from the Department of Bacteriology, National Institute for Research in Tuberculosis. Viability of all the isolates was maintained on LJ slopes. High titre of mycobacteriophage phAE129 used in this study was prepared using* M. smegmatis* mc^2^155 in Middlebrook 7H9 complete medium [17].

About 350 *μ*L of G7H9 broth supplemented with 10% albumin dextrose complex and 0.5% glycerol was taken in cryovials and added with 50 *μ*L of crude extract in order to get the final concentration of 100 *μ*g/mL. 100 *μ*L of* M. tuberculosis* cell suspension was added to all the vials. The above procedure was followed for all the three* M. tuberculosis* isolates. DMSO (1%) was also included in the assay as solvent control. All the vials were incubated at 37°C for 72 h. After incubation, 50 *μ*L of high titre phage phAE129 and 40 *μ*L of 0.1 M CaCl_2_ solution were added to the test and control vials. All the vials were incubated at 37°C for 4 h. After incubation, 100 *μ*L from each vial was transferred to luminometer cuvette. About 100 *μ*L of D-Luciferin was added and relative light unit (RLU) was measured in luminometer (Monolight 2010):

(1) Percentage  RLU  reduction  =Control  RLU−Test  RLUControl  RLU×100.

Extracts showing RLU reduction by 50% or more when compared to control were considered as having antitubercular activity. Actinomycetes which showed activity against all the three* M. tuberculosis* isolates were selected and tested in triplicate against the same* M. tuberculosis* isolates and nonmycobacterial pathogens. The mean values of the results were calculated.

### 2.4. In Vitro Screening against Nonmycobacterial Pathogens

Test pathogens used in this study include standard strains* Staphylococcus aureus* MTCC96,* Bacillus subtilis* MTCC441, clinical isolates of* Escherichia coli*,* Salmonella typhi*,* Klebsiella pneumoniae*,* Proteus vulgaris*,* Pseudomonas aeruginosa*, and* Candida albicans*. All the nonmycobacterial pathogens were obtained from the Department of Microbiology, Periyar University, Tamil Nadu. Test pathogens were inoculated onto Muller Hinton Agar (MHA) plates using sterile cotton swab. Actinomycete cultures were inoculated onto YEME agar plates (20 mL/plate) and incubated at 28°C for 10 d. After scraping the mycelial growth, 5 mm diameter agar plugs were taken and placed over MHA plates seeded with test pathogens. All the plates were incubated at 37°C for 24 h for bacteria and 48 h for fungi. Agar plug prepared from uninoculated YEME agar was included as medium control. Zone of inhibition was expressed in millimetre in diameter [15].

## 3. Results

### 3.1. Isolation and Characterization of Actinomycetes

Seventy-two actinomycete isolates were isolated from soil samples collected from different ecosystems. Based on the microscopic and cultural characteristics, 54 different actinomycete isolates were selected after dereplication of 18 isolates. Fifty out of 54 isolates produced good growth with powdery consistency on YEME agar. Majority of the isolates produced either white (48%) or gray (37%) colour aerial mycelium. Reverse side colour and colour of soluble pigments produced by these actinomycetes include brown, yellow, pink, green, blue, and orange. In micromorphological study, except the strain S28, all the actinomycete isolates showed the presence of substrate and aerial mycelium with different length and arrangement. In 34 (62.96%) isolates, the aerial mycelium was of rectiflexibile (RF) type (Table 1). Based on the observed phenotypic characteristics, 43 actinomycete isolates (79.62%) were tentatively identified as* Streptomyces* sp. and 11 (20.37%) isolates were identified as nonstreptomyces/rare actinomycetes.

### 3.2. Production and Antitubercular Activity of Actinomycete Extracts

All the actinomycete isolates showed growth on YEME agar medium. Overall about 30–40 mg of crude extract was obtained from 50 mL of YEME agar medium inoculated with actinomycetes. Extracts from 39 (72.22%) out of 54 isolates inhibited one or more of* M. tuberculosis* isolates tested. Extracts of 16 (29.62%) actinomycete isolates inhibited the growth of all the three strains, namely, standard strain* M. tuberculosis* H37Rv, SHRE sensitive, and SHRE resistant* M. tuberculosis* isolates. Extracts of 24 (44.44%) actinomycetes inhibited the standard strain* M. tuberculosis* H37Rv, whereas the SHRE sensitive and SHRE resistant* M. tuberculosis* isolates were inhibited by the extracts of 23 (42.59%) actinomycetes (Table 2).

The diversity of antagonistic activity exhibited by the actinomycetes is given in Table 3. In total, 43 (79.63%) actinomycetes showed antagonistic activity against one or more of the 11 pathogens tested. Eleven (20.37%) actinomycetes failed to inhibit any of the test pathogens. Another eleven isolates (20.37%) were active against one or more number of* M. tuberculosis* isolates, Gram positive bacteria, and Gram negative bacteria. Eight (14.81%) isolates were active only against Gram positive bacteria and* M. tuberculosis* isolates whereas one each exclusively inhibited Gram positive bacteria (Y1), Gram negative bacteria (M17), fungi (CSA19), Gram positive and Gram negative bacteria (Y32), and* M. tuberculosis* and Gram negative bacteria (M7). Only two isolates, K8 and TA36, inhibited both* M. tuberculosis* isolates and* C. albicans* without showing activity against any other bacterial pathogens. The only strain CSA15, isolated from coffee plantation soil, showed activity against all the* M. tuberculosis* isolates, Gram positive and Gram negative bacteria and fungi. Thus, diverse antagonistic activity was exhibited by the actinomycetes isolated from all the rare ecosystems investigated in this study.

### 3.3. Antagonistic Activity against Nonmycobacterial Pathogens

In agar plug method, 27 isolates, that is, 50% of the actinomycete isolates, showed inhibition against at least one or more number of nonmycobacterial pathogens tested. Maximum of 17 (31.48%) and 16 (29.62%) isolates were active against* S. aureus* MTCC96 and* B. subtilis* MTCC441, respectively. Among the Gram negative bacteria,* P. vulgaris* was inhibited by 11 (20.37%) actinomycetes. None of the isolates were found to be active against* P. aeruginosa*. Four (5.55%) actinomycetes inhibited the growth of* C. albicans* (Table 2). Ten out of 18 soluble pigment producers were found to exhibit antibacterial activity. The crude ethyl acetate extracts from 16 selected actinomycete isolates showed activity against the bacterial pathogens and* M. tuberculosis* strains tested. The mean value of activity is presented in Table 4.

## 4. Discussion

Pathogenic microorganisms have evolved sophisticated mechanisms to inactivate antibiotics and rendered an urgent need for new antibiotics that would target the emerging multidrug resistance [18]. Consequently, search for novel sources of potent antibiotics is desperately needed to develop potent drugs. Microbial resources have made an incredible contribution to the antibiotic drug discovery and development process over the last seven decades [19]. In particular, actinomycetes are the most important source of bioactive natural compounds with a long track record of producing novel molecules [20]. The present study reported the bioactive potential of actinomycetes isolated from less explored ecosystems against* M. tuberculosis* and other nonmycobacterial pathogens.

According to Bérdy [7] great number of antibiotic compounds exhibit exclusive activities against Gram positive bacteria while only 1.5% are active against Gram negative bacteria. In the present study, 50% of the actinomycetes isolated from rare ecosystems showed activity against nonmycobacterial pathogens, whereas 72% of the isolates are found to inhibit sensitive and/or resistant strains of* M. tuberculosis*. Similar to earlier reports [21, 22] more number of actinomycetes showed inhibited Gram positive bacteria compared to Gram negative bacteria.

Wide variation in the percentage of active isolates and in their activity spectra has been reported from different ecosystems [6, 22, 23]. Eleven different patterns of activities were recorded against 11 test organisms. The key observations made in this analysis include the following: (i) antagonistic isolates are distributed in all the ecosystems studied; (ii) the maximum number of isolates (29.62%) is exclusively active against* M. tuberculosis* followed by 20.37% of the isolates inhibiting Gram positive and Gram negative bacteria and* M. tuberculosis*. The broad spectrum of activity detected in some of the* Streptomyces* isolates in this study could be due to different antimicrobial compounds produced by the isolates, each one with a species- or group-specific activity [22] and/or to the presence of more than one compound with broad spectrum of action like the antibiotic meroparamycin which is active against Gram positive bacteria, yeasts, and filamentous fungi [24], among others. These findings clearly evidenced that antagonistic actinomycetes isolated from rare ecosystems are potential sources for compounds showing antibacterial and antituberculous activity.

Certain issues are associated with primary screening in solid medium and liquid culture based secondary screening. There can be loss of bioactive metabolite production in secondary screening while using a medium which is different from the one used for primary screening. Certain actinomycete strains produce antibiotic compounds only in solid medium but fail to produce the same in liquid medium [25–27]. Moreover, the whole organism based primary screening methods like cross streak/cross spot methods are not suitable for highly biohazardous organisms like* M. tuberculosis*. To address all these issues, agar plugs which contain the extracellular bioactive compounds were tested against nonmycobacterial pathogens. The crude extract from the same medium was tested against* M. tuberculosis*. Most of the secondary metabolites including antibiotics are extracellular in nature and extracellular products of actinomycetes exhibit potent antimicrobial activities [28–31]. Similar observations were made in the present study.

There are number of mycobacterial drug susceptibility assays used for the screening of natural products described over the period of time [32]. Screening methods using LJ or 7H11 agar require large amounts of crude extracts or purified compounds to be incorporated in the media. It also requires about three or more weeks of incubation to produce results. Conventional testing using LJ slants is unsuitable for screening uncharacterised novel compounds whose heat stability is unknown. LRP assay used in this study is a rapid, liquid culture based and less laborious method for high throughput screening of a large number of compounds for antimycobacterial activity [33]. As a broth based method, LRP assay qualifies to be ideal for screening such novel compounds. The need for only small quantity of the extracts/compounds to obtain results within 3 days qualifies the test further. Natural products from various natural sources like plants, actinomycetes, and fungi [16, 34, 35] and synthetic compounds [36] had been screened for antitubercular activity by adopting LRP assay.

Antibiotics isolated and characterised from strains that show broad spectrum activity can be used to treat varied microbial infections. The availability of such drugs across the counter and wide usage of the same may lead to the emergence and spread of drug resistance among the existing pathogens [37]. Selection of antibiotics that are exclusively acting against* M. tuberculosis* and/or limited number of nonmycobacterial pathogens may circumvent this problem.

## 5. Conclusion

Findings of the present work concluded that less explored ecosystems investigated in this study are the potential resource for bioactive actinomycetes. However, in this study, only the crude extracts were evaluated and found to be active against* Mycobacterium tuberculosis* and other nonmycobacterial pathogens. In general, crude extracts are complex mixture of compounds. So additional research like bioassay guided fractionation and characterization will be needed to validate whether a single useful compound can be found and it is also needed to determine the MIC and meaningful toxicity and specificity studies. In particular, isolation and characterization of active molecule from the potential strains like D25, CSA15 will pave the way for the development of promising antibiotics against* M. tuberculosis* and other nonmycobacterial pathogens.

## Figures and Tables

**Table 1:** Growth and morphological pattern of actinomycetes isolated from different rare ecosystems.

Characteristics	Appearance	Number of isolates (%)
Growth	Good	50 (92%)
	Moderate	4 (7%)

Consistency	Powdery	45 (83%)
	Leathery	9 (16%)

Aerial mass colour	White	26 (48%)
	Gray	20 (37%)
	Green	2 (3%)
	Blue	1 (2%)
	Orange	2 (3.70%)
	Pink	3 (5.55%)

Reverse side pigment		17 (31.48%)

Soluble pigment		18 (33.33%)

Micromorphology	Aerial and substrate mycelium	53 (98.14%)
	Substrate mycelium alone	1 (1.85%)

Spore chain morphology	Rectus flexible (RF)	34 (62.96%)
	Retinaculum apertum (RA)	6 (11.11%)
	Spirals (S)	2 (3.70%)
	Others	12 (22.22%)

**Table 2:** Number of actinomycetes exhibiting activity against nonmycobacterial, fungal, and mycobacterial pathogens.

Ecosystems	Number of actinomycetes	Number of antagonistic isolates against										
		Nonmycobacterial pathogens							*M. tuberculosis *			Fungi
		*S. aureus *MTCC96	*B. subtilis *MTCC 441	*E. coli *	*S. typhi *	*P. vulgaris *	*K. pneumoniae *	*P. aeruginosa *	*MTB H37Rv *	*MTB SHRE sensitive *	*MTB SHRE resistant *	*C. albicans *
Thar Desert, Rajasthan	5	4	3	—	—	3	—	—	5	5	5	—
Rubber forest, Kerala	6	1	1	—	—	1	—	—	2	3	1	1
Coffee plantation, Kerala	12	3	1	1	2	2	2	—	5	5	5	2
Thirukurungudi, Tamil Nadu	7	3	—	—	—	2	—	—	3	4	2	1
Yercaud Hills, Tamil Nadu	9	4	4	—	1	1	1	—	4	1	4	—
Thottabetta Hills, Tamil Nadu	3	—	3	—	—	—	1	—	2	2	3	—
Munnar Hills, Tamil Nadu	5	—	1	—	—	3	—	—	3	3	3	—
Siruvani Hills, Tamil Nadu	7	2	3	1	1	—	—	—				—

Total Isolates	54	17	16	2	4	11	3	—	24	23	23	4

Percentage	100	31.48	29.62	3.7	7.4	20.37	5.55	0.00	44.44	42.59	42.59	5.55

**Table 3:** Diversity of antagonistic activity exhibited by actinomycetes isolated from rare ecosystems.

Actinomycetes active only against	Actinomycetes from different ecosystems								Total active isolates	Percentage
	Thar desert	Rubber forest	Coffee plantation	Thirukurungudi	Yercaud hills	Thottabetta	Munnar hills	Siruvani hills		
*M. tuberculosis *	D18	K11, K38	CSA2, CSA4, CSA20	TA4, TA27	Y3, Y7, Y13, Y23, Y25	—	M2, M3	S8	16	29.62
G+ bacteria	—	—	—	—	Y1	—	—	—	1	1.85
G− bacteria	—	—	—	—	—	—	M17	—	1	1.85
Fungi	—	—	CSA9	—	—	—	—	—	1	1.85
MTB, G+ and G− bacteria, and fungi	—	—	CSA15	—	—	—	—	—	1	1.85
MTB and G+ and G− bacteria	D6, D13, D16	K3	CSA24	TA3, TA22	Y10	T2	M8	S2	11	20.37
MTB and G+ bacteria	D25	—	CSA19	TA38	Y8	T6, T7	—	S4, S5	8	14.81
MTB and G− bacteria	—	—	—	—	—	—	M7	—	1	1.85
G+ and G− bacteria	—	—	—	—	Y32	—	—	—	1	1.85
G+ and G− bacteria and fungi	—	—	—	—	—	—	—	—	—	0.00
MTB and fungi	—	K8	—	TA36	—	—	—	—	2	3.70

Total number of active isolates	5	4	7	6	9	3	5	4	43	79.63

No activity	—	K7, K10	CSA7, CSA8, CSA11, CSA12, CSA18	TA34	—	—	—	S21, S26, S28	11	20.37

Total isolates	5	6	12	7	9	3	5	7	54	100

**Table 4:** Activity of selected actinomycetes against bacteria, fungi, and *M. tuberculosis *isolates.

Actinomycetes	*S. aureus * *MTCC96 *	*B. subtilis* *MTCC441 *	*E. coli *	*S. typhi *	*P. vulgaris *	*K. pneumoniae *	*P. aeruginosa *	*C. albicans *	*M. tuberculosis H37Rv *	SHRE sensitive *M. tuberculosis *	SHRE resistant *M. tuberculosis *
D6	12.3 ± 0.5	13 ± 0.00	—	—	15.6 ± 0.52	—	—	—	69.6 ± 2.5	74.64 ± 2.55	62.42 ± 2.78
D13	13.2 ± 0.6	—	—	—	12.2 ± 0.34	—	—	—	95.13 ± 2.1	93.65 ± 2.53	83.42 ± 3.65
D16	11.5 ± 0.5	10.5 ± 0.5	—	—	8 ± 0.00	—	—	—	49.86 ± 2.1	75.31 ± 3.61	65.06 ± 2.58
D18	—	—	—	—	—	—	—	—	86.41 ± 1.8	73.79 ± 3.46	95.04 ± 3.16
D25	21.1 ± 0.2	22.6 ± 0.59	—	—	—	—	—	—	87.10 ± 2.6	75.81 ± 2.29	80.71 ± 3.24
K38	—	—	—	—	—	—	—	—	77.05 ± 1.5	68.12 ± 2.43	75.46 ± 3.80
CSA15	10.5 ± 0.5	8.3 ± 0.40	10.3 ± 0.26	12.1 ± 0.5	10.8 ± 0.41	12 ± 0.0	—	10.6 ± 0.3	85.98 ± 0.9	94.72 ± 3.66	67.37 ± 2.74
CSA20	—	—	—	—	—	—	—	—	73.72 ± 2.9	74.89 ± 2.43	79.40 ± 2.93
TA3	11 ± 0.00	—	—	—	7.86 ± 0.11	—	—	—	65.0 ± 2.29	91.56 ± 3.51	53.47 ± 2.22
TA27	—	—	—	—	—	—	—	—	81.29 ± 3.2	94.92 ± 3.06	80.39 ± 3.17
T6	—	22.3 ± 0.55	—	—	—	—	—	—	79.26 ± 0.4	86.2 ± 2.36	96.21 ± 3.32
T7	—	20.5 ± 0.50	—	—	—	—	—	—	91.58 ± 4.5	83.32 ± 3.50	93.44 ± 3.50
M7	—	—	—	—	9.3 ± 0.26	—	—	—	67.3 ± 2.33	96.38 ± 1.28	69.66 ± 4.16
M8	—	10.4 ± 0.43	—	—	14 ± 0.00	—	—	—	56.8 ± 2.02	66.31 ± 1.21	58.11 ± 1.84
S2	—	15 ± 0.00	7 ± 0.00	7.2 ± 0.3	—	—	—	—	93.48 ± 3.0	83.16 ± 2.83	91.093 ± 4.55
S4	12.1 ± 0.2	21.6 ± 1.02	—	—	—	—	—	—	96.16 ± 3.1	88.62 ± 3.01	67.326 ± 2.31
